# Optimization of Magnetic Biochar Preparation Process, Based on Methylene Blue Adsorption

**DOI:** 10.3390/molecules29215213

**Published:** 2024-11-04

**Authors:** Bin Liu, Yixuan Wu, Zebing Xing, Ji Zhang, Yuxin Xue

**Affiliations:** 1 College of Agricultural Engineering, Shanxi Agricultural University, Jinzhong 030801, China; 20233058@stu.sxau.edu.cn (B.L.); zhangji9171018@163.com (J.Z.); 18235488991@163.com (Y.X.); 2 College of Food, Shanxi Agricultural University, Jinzhong 030801, China; dwu4008@163.com

**Keywords:** mushroom substrate, magnetic biochar, process optimization, adsorption

## Abstract

The search for low-cost and effective adsorbents for the removal of organic dyes from contaminated water is urgently needed. The substantial amount of waste mushroom cultivation substrates generated in practical production can serve as an ideal material for the preparation of adsorbents. In this study, we investigated the main control parameters affecting the performance of magnetic mushroom substrate biochar and optimized the process of preparing biochar by using the Plackett–Burman and central composite design methods. Various analytical techniques including SEM, EDX, BET, and VSM were used to characterize the biochar. The results indicate that the carbonization temperature had the most significant impact on the yield and adsorption performance of biochar. Under the conditions of a carbonization temperature of 600 °C, a carbonization retention time of 1 h, and an impregnation ratio of 0.1, the yield and methylene blue adsorption value of magnetic biochar were 42.54% and 2297.04 μg/g, respectively, with a specific surface area of 37.17 m^2^/g. This biochar effectively removed methylene blue from the solution, demonstrating a high economic efficiency for wastewater treatment and pollution control. Furthermore, the adsorption–desorption cycle studies revealed its excellent stability and reusability. Additionally, based on the response surface methodology, a three-dimensional surface model of the adsorption performance of magnetic biochar under different carbonization conditions was established, providing a theoretical basis for the preparation of magnetic biochar from agricultural wastes.

## 1. Introduction

Organic dyes are commonly used color additives that are widely applied in industries such as textiles, inkjet printing, paper manufacturing, and cosmetics. However, industrial production inevitably results in wastewater containing organic dye pollutants [1,2,3]. For instance, methylene blue (MB) is a typical water-soluble dye compound with extensive industrial applications. Organic dye pollutants possess strong toxicity and carcinogenicity. When wastewater containing these pollutants is discharged directly into the environment, it poses significant threats to human health and the survival safety of other organisms. Therefore, the removal of these organic dyes from wastewater before discharge has become an urgent issue to address [4,5].

Biochar is a black, carbon-rich, porous solid material produced by performing the thermochemical conversion of biomass in the absence of oxygen [6,7]. It is characterized by a large specific surface area, large pores, rich functional groups, and abundant organic carbon and mineral content. These properties enable biochar to interact with pollutants in aqueous solutions and soil through pore filling, π-π interactions, surface complexation, hydrogen bonding, and electrostatic forces [8], making biochar an effective adsorbent for water pollution control, soil remediation, and agricultural development. Its good physicochemical properties, well-developed internal pores, and strong adsorption ability have garnered increasing attention [9].

However, powdered biochar’s small particle size and low density make it difficult to separate it from the solution post-use. Modifying biochar with ferromagnetic elements (Fe, Co, and Ni) and their oxides enables recovery based on external magnetic fields, reducing time and costs [10,11]. The physicochemical properties and adsorption ability of magnetic biochar are influenced by the preparation method employed, where common ones for iron-based biochar composites include ball milling [12,13], chemical coprecipitation [14], impregnation pyrolysis [15], hydrothermal synthesis [16,17], and liquid-phase reduction [18]. For example, a recent study showed that a pyrolysis–hydrothermal synthesis method can produce a notably large surface area (749.10 m^2^/g) and endow CPAC@-Fe_3_O_4_ with strong mesoporous characteristics; this method was demonstrated to have high 2,4-dichlorophenoxyacetic acid adsorption ability (160.71 mg/g) [19]. Magnetic biochar with Fe_3_O_4_ and Fe_2_O_3_ and a rougher surface, sharper corners and edges, and a more compact, regular pore structure had the maximum Cr (VI) adsorption ability (83.02 mg/g) and was obtained with electromagnetic induction pyrolysis [20]. Among the commonly used modification methods, impregnation pyrolysis is particularly advantageous due to its simplicity in terms of operation, low costs, and suitability for large-scale industrial production. Moreover, the magnetic biochar prepared with this method exhibits high saturation magnetization and superparamagnetic behavior [21,22], where the latter enables simple, rapid, and effective separation, making magnetic biochar adsorbents a promising solution [23].

Cultivation substrate that is not completely absorbed by edible mushrooms is referred to as mushroom substrate. According to calculations, the production of 1 kg of fresh edible mushrooms generates about 5 kg of mushroom substrate by-products [24]. In 2022, the production of edible mushroom substrate in China exceeded 200 million tons [25]. Currently, in China, the utilization rate of edible mushroom substrates is relatively low, with many growers in this sector incinerating, disposing of, crushing, and returning mushroom substrates to the field post-harvest [26]. This generates large quantities of bacteria, molds, and pests, polluting the environment and posing safety risks to humans and animals [27]. Therefore, as one of the primary by-products of edible mushroom cultivation, mushroom substrate can be utilized to prepare biochar materials, thereby preventing pollution and resource waste.

The carbonization process is usually based on a one-factor test for the analysis of the effects of individual process parameters while other factors remain unchanged. However, this requires numerous experiments [28] and precludes the observation of interactions among factors, hindering a comprehensive analysis of the carbonization process. In order to obtain an optimized solution for the preparation of magnetic biochar, a more convenient experimental design approach with which we can effectively adjust process parameters and reduce production costs is needed. Among the available techniques, the Plackett–Burman (P-B) and response surface–central composite (RSM-CCD) methods are often used for efficient experimental design and process optimization [29]. The P-B design is a cost-effective and efficient two-level experimental design where the significance of the factors is determined by comparing the difference between two levels of each factor and the overall difference. Factors with significant influence are thus identified, achieving the screening aims and avoiding the waste of experimental resources due to the presence of too many or insignificant factors in subsequent optimization tests [30]. It has been shown that RSM-CCD can be used to obtain optimal results in multi-factor tests and is widely used in agriculture, biology, and other fields [31]. Response surface methodology, a multivariate statistical method, involves the use of second-order polynomial equations to interpolate experimental data for prediction. The desired optimal conditions can be determined by evaluating the interactions among the parameters [32].

Accordingly, in this study, we investigated the production of biochar from *pleurotus ostreatus* substrate as a green and sustainable method for synthesizing magnetic biochar by using the impregnation pyrolysis process. Moreover, the Plackett–Burman (P-B) and response surface methodology–central composite design (RSM-CCD) were employed to optimize the experimental conditions, revealing the influence of and interactions among factors. This investigation aimed to explore the capability of this composite to degrade the MB dye, thus addressing the broader imperative of eco-friendly and enduring water treatment solutions.

## 2. Experimental Process and Analysis

### 2.1. Experimental Condition Design

Under the premise of a carbonization power of 700 w (Table 1), the effects of four factors—carbonization temperature (A), carbonization retention time (B), m(Fe^3+^):m(POS) (C), and feedstock particle size (D)—on the yield and adsorption properties of magnetic *pleurotus ostreatus* substrate biochar (MPOSB) during pyrolysis were investigated. The influence of the first three factors on the MPOSB adsorption properties was also analyzed.

The experiment was initially completed by using a P-B experimental design in Design-Expert 13 software, setting two levels of (high (+1) and low (−1)) for each response variable, as shown in Table 2. The experimental response values, including the yield (Y_1_) and the methylene blue adsorption value (S_1_) of MPOSB, were determined.

With P-B design experimental analysis, it was found that the effects of carbonization temperature, carbonization retention time, and m(Fe^3+^):m(POS) on the MPOSB yield were extremely significant and that all four factors considered had extremely significant effects on the adsorption performance of MPOSB. Moreover, the adsorption performance of the MPOSB whose particle size was 0.1–0.45 mm was significantly higher than that of the MPOSB whose particle size was 0.45–1 mm. To further investigate the interaction effects of each response variable on the MPOSB adsorption performance, a three-factor, three-level CCD experimental design was adopted. The particle size of the raw material used was 0.1–0.45 mm, and the carbonization temperature, carbonization retention time, and m(Fe^3+^):m(POS) were labeled A, B, and C, respectively. The response variables were designed at the three levels of −1, 0, and +1, as shown in Table 3.

### 2.2. P-B Experimental Analysis

The MPOSB yield and methylene blue adsorption value were calculated according to the P-B experimental design, and 12 sets of experimental response values were obtained, as shown in Table 4. Seven groups of dummy variables (E, F, G, H, J, K, and L) were included in the experimental design to investigate possible errors during the experiment.

To analyze the significance of each response variable’s effect on the response values, the response values in Table 4 were regressed. The following regression equations were obtained for the MPOSB yield *Y*_1_ (1) and the methylene blue adsorption value *S*_1_ (2):

(1) $$Y_{1}=78.02-3.13A-0.733B+0.7833C-0.2333D\;\left(99.93\%\right),$$

(2) $$S_{1}=1394.07+192.15A-82.82B-124.95C-429.92D\;\left(99.93\%\right),$$

We easily ascertained that the regression equation adequately fit the experimental data. The results of the deviation regression coefficients and significance tests are shown in Table 5. The F- and *p*-values are the hypothesis test values indicating the significance of the response variables’ effect on the response values [33].

Based on the significance of the response variables’ effect on the MPOSB yield, we found that the *p*-values of carbonization temperature, carbonization retention time, and m(Fe^3+^):m(POS) were all less than 0.0001, indicating their extremely significant effects on the MPOSB yield. By also considering the magnitude of the F-value, it could be inferred that the magnitudes of the effects of the three response variables on the MPOSB yield were ordered as follows: carbonization retention time < m(Fe^3+^):m(POS) < carbonization temperature. The *p*-value of the raw material particle size was less than 0.001, indicating that it had a relatively significant effect on the MPOSB yield.

From the significance of the response variables’ effect on the adsorption performance of the MPOSB, it was calculated that the *p*-values of carbonization temperature, carbonization retention time, m(Fe^3+^):m(POS), and raw material particle size were all less than 0.0001, indicating that they all had extremely significant effects on the adsorption performance of the MPOSB. The magnitudes of these effects were found to be in the following order: carbonization retention time < m(Fe^3+^):m(POS) < carbonization temperature < raw material particle size. By also considering Table 4, it was ascertained that the adsorption performance of the MPOSB whose particle size was 0.1–0.45 mm was significantly better than that of the MPOSB whose particle size was 0.45–1 mm. This might have been due to the relatively small specific surface area of the latter, resulting in larger internal diffusion resistance and lower adsorption affinity [34]. Therefore, the particle size of the raw material of biochar should not be too large.

### 2.3. CCD Experimental Analysis

CCD tests were conducted for carbonization temperature, carbonization retention time, and m(Fe^3+^):m(POS) at feedstock particle sizes of 0.1–0.45 mm to determine the effect of the interaction of the response variables on the response values during pyrolysis. Based on the design in Table 3, three-factor, three-level MPOSB yield, and adsorption performance tests were completed, and 20 sets of data were obtained, as shown in Table 6.

#### 2.3.1. MPOSB Methylene Blue Adsorption Values

The regression analysis of the MPOSB methylene blue adsorption response values, which are presented in Table 5, was performed by using the CCD method, and the regression equation for the adsorption value (*S*_2_) model was obtained (3) as

(3) $$S_{2}=1035.59+215.84A-22.48B-98.31C-30.61AB+55.61AC-\;55.11BC-523.5A^{2}+133.62B^{2}+197.67C^{2}\;\left(R_{1}^{2}=95.35\%\right),$$

where A, B, and C represent the carbonization temperature, the carbonization retention time, and m(Fe^3+^):m(POS), respectively. The positive and negative symbols in the regression equations indicate synergistic and antagonistic effects, respectively. $R_{1}^{2}$ is the multivariate correlation coefficient, reflecting the model correlation degree, where the larger the $R_{1}^{2}$, the better the correlation [35].

The regression equation was analyzed with ANOVA (Table 7), where the F- and *p*-values are the hypothesis test values indicating the response variables’ significance on the response values. According to the test of the MPOSB methylene blue adsorption significance, the model *p*-value was <0.0001, and the F-value was 22.77, indicating highly significant predictions. The $R_{1}^{2}$ of the model was 0.9535, indicating that it could better reflect response value changes at the 95.35% level [36]. The model could better explain the relationship among the MPOSB methylene blue adsorption and carbonization temperature, carbonization retention time, and m(Fe^3+^):m(POS).

**Figure 1:**
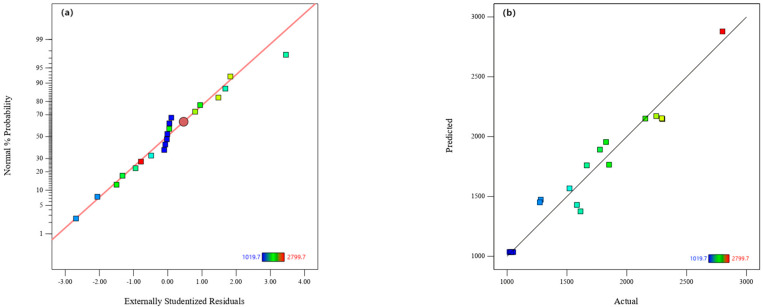
Validation of MPOSB adsorption values. (**a**) Normal plot of residual values. (**b**) Comparison of predicted and actual values.

The normal probability distribution of the MPOSB methylene blue adsorption residuals is plotted in Figure 1a. The data points were nearly linear, indicating that the model accurately described the relationship between the response variables and values, which indicated that it was well adapted [37]. The distributions of the predicted and actual values are shown in Figure 1b, where the actual values are plotted more densely near the prediction line, indicating a good model fit. Therefore, the prediction model could effectively predict the adsorption ability of the MPOSB.

#### 2.3.2. MPOSB Yield

Similarly, the MPOSB yield response values, shown in Table 6, were analyzed to obtain the regression equation for the yield (*Y*_2_) model (4) as

(4) $$Y_{2}=45.38-4.25A-0.4323B+1.43C-0.475AB-0.15AC+0.938A^{2}-\;0.0694B^{2}-0.5998C^{2}\;\left(R_{2}^{2}=95.27\%\right),$$

where *A*, *B*, and *C* represent the carbonization temperature, the carbonization retention time, and m(Fe^3+^):m(POS), respectively. The positive and negative symbols in the regression equations indicate synergistic and antagonistic effects, respectively. $R_{2}^{2}$ is the multivariate correlation coefficient of the equations, reflecting the degree of model correlation, where the larger the $R_{2}^{2}$, the better the correlation.

**Figure 2:**
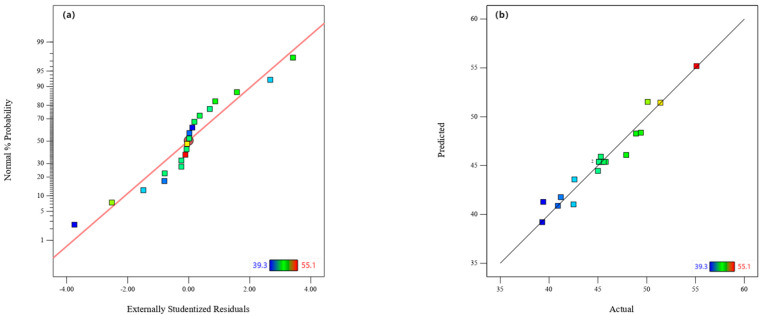
Validation of MPOSB yield. (**a**) Normality plot of residual values. (**b**) Comparison between predicted and actual values.

As shown in Table 8, ANOVA was used to test the accuracy of the model for the MPOSB yield Y_2_. The assumed significance value, F = 22.4, with *p* < 0.0001, proved that the model had extremely significant features. The main interactions of carbonization temperature and carbonization retention time, carbonization temperature and m(Fe^3+^):m(POS), and carbonization retention time and m(Fe^3+^):m(POS) were not significant, while the main interaction terms of carbonization temperature and m(Fe^3+^):m(POS) were significant. Further, the main and secondary interaction terms of carbonization retention time and the secondary interaction terms of carbonization temperature and m(Fe^3+^):m(POS) did not significantly affect the target response values. The model regression coefficient was 0.9527, indicating that the model better reflected the changes in response values at the 95.27% level, i.e., it was in good agreement with the actual data, and the interaction between the response values and the three response variables was well reflected. The method is feasible and reliable for the predictive analysis of the MPOSB yield. The plots of residual distribution and predicted distribution are shown in Figure 2. Again, the distribution of the data points in Figure 2a was nearly linear, with the same error in both directions, indicating that the model had good normality. As shown in Figure 2b, the actual values were uniformly distributed above and below the predicted line and were closely stacked. This strongly supports the accuracy of the multi-factor interaction relationship obtained with the CCD experimental design method.

### 2.4. Carbonization Process Optimization Analysis

**Figure 3:**
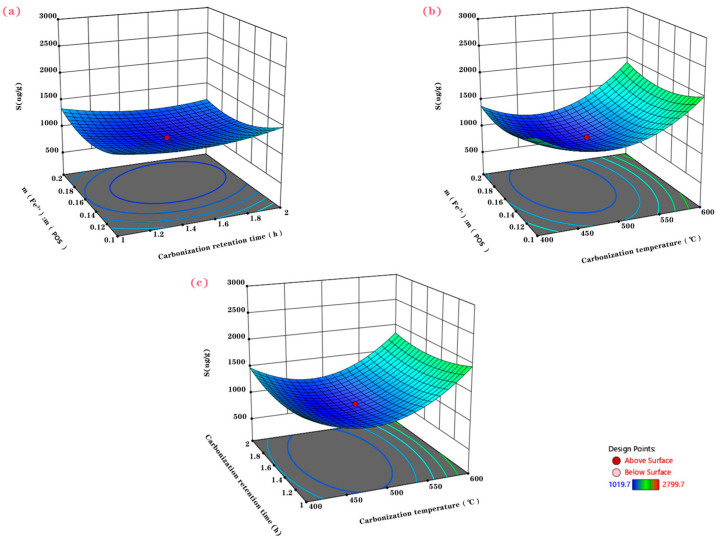
Effects on MPOSB adsorption values with different carbonization parameters. (**a**) Interaction between carbonization retention time and m(Fe^3+^):m(POS) at carbonization temperature of 500 °C. (**b**) Interaction between carbonization temperature and m(Fe^3+^):m(POS) at carbonization retention time of 1.5 h. (**c**) Interaction of carbonization temperature and carbonization retention time when m(Fe^3+^):m(POS) was 0.15.

The effects of the three response variables on the adsorption performance of the MPOSB were analyzed by using the response surface methodology, as shown in Figure 3. The S-values no longer monotonically increased or decreased with a single parameter but varied with the interactions among multiple response variables. Figure 3c shows the variation curves of the MPOSB adsorption values with the change in carbonization temperature and retention time when m(Fe^3+^):m(POS) was set to 0.15. Within the interaction range of these two response variables, the MPOSB adsorption performance decreased and then increased with the increase in the carbonization temperature, and the lowest adsorption value was recorded at a carbonization temperature of 500 °C. The reasons for this might be threefold. Firstly, this might be due to the fact that under oxygen-limited conditions, as the pyrolysis temperature increased, the inorganic iron salts first underwent chloritization and hydroxylation, forming various hydrogenated iron oxides with different abundance rates. Subsequently, at higher temperatures, they underwent dehydration and transformed into a strong magnetic maghemite, which was further heated to be structurally rearranged into a weak magnetic hematite. The hematite then reacted with the carbon formed through carbonization and transformed into magnetite, which was strongly magnetic. Some of these could also be reduced to pure metals by the strong reducing gases produced during pyrolysis as the temperature increased again [38]. Secondly, this result might have been due to the high heating rate induced by rapid pyrolysis, which promoted the thermal cracking of biomass, leading to the production of liquids and volatiles. Additionally, this process also generated a higher content of tar, which was not conducive to the contact between the carbon materials and the external environment, thereby reducing the effective specific surface area [39]. Thirdly, it might be that the positively charged quaternary ammonium groups in the molecular structure of methylene blue inhibited the biochar’s methylene blue adsorption ability when the inorganic iron salt was present in the ionic state [40]. Similar results were obtained, as can be observed in Figure 3b, when the carbonization retention time was kept constant. The adsorption values slightly decreased and then increased as the carbonization retention time and m(Fe^3+^):m(POS) changed, indicating that the interaction between carbonization retention time and m(Fe^3+^):m(POS) did not have a significant effect on the adsorption ability of the MPOSB.

## 3. Process Optimization Results

An optimization analysis was performed with Design-Expert 13 software to determine the suitability of the carbonization process, where the closer to 1 the desirability value is, the more suitable the process conditions for preparing the MPOSB meeting adsorption performance and yield requirements are [41]. According to the analysis of the optimization model data, when the carbonization temperature, carbonization retention time, and the m(Fe^3+^):m(POS) ratio were set to 600 °C, 1 h, and 0.2, respectively, the model exhibited a methylene blue adsorption value of 2171.711 μg/g, and the predicted yield of MPOSB was 43.582%. Under these process conditions, the model achieved the highest predicted adsorption value. When these three factors were set to 400 °C, 1 h, and 0.2, respectively, the modeled MPOSB yield was 51.438%, while the predicted adsorption value of methylene blue was 1567.584 μg/g, representing the maximum predicted MPOSB yield under these process conditions. Since the interaction between the carbonization retention time and the m(Fe^3+^):m(POS) value did not significantly affect the adsorption ability (Figure 3a), the carbonization process parameters were adjusted to prepare two groups of MPOSB—index X_1_ and index X_2_—suitable for production. For index X_1_, the model-predicted methylene blue adsorption value and yield of MPOSB were 2146.887 μg/g and 41.027%, respectively, when the carbonization temperature, carbonization retention time, and m(Fe^3+^):m(POS) value were 600 °C, 1 h, and 0.1, respectively. Compared with the model-predicted maximum adsorption values (600 °C, 1 h, and 0.2), the methylene blue adsorption value and yield of biochar only decreased by 1.14% and 6.55%, respectively. For index X_2_, when the carbonization temperature, carbonization retention time, and m(Fe^3+^):m(POS) values were set to 400 °C, 1 h, and 0.1, respectively, the model-predicted methylene blue adsorption value and yield of MPOSB were 1765.21 μg/g and 48.283%, respectively. Compared with the model-predicted value of the maximum yield (400 °C, 1 h, and 0.2), the adsorption value of methylene blue increased by 12.61%, while the yield decreased by 6.13%, respectively. The trade-off between the MPOSB yield and adsorption performance according to indices X_1_ and X_2_ is an economic consideration in the production process of MPOSB, and optimizing the m(Fe^3+^):m(POS) value from 0.2 to 0.1 could effectively reduce the production costs. The results of the MPOSB prepared under four sets of process conditions for validation tests are shown in Table 9. According to the comparison of the target values with the experimental test values, most of the relative errors were within 5%, verifying the applicability of the optimization model.

To validate the optimization of the carbonization process for the MPOSB adsorption performance (Table 9), we analyzed the specific surface area of the four MPOSB types, as shown in Table 10. Due to the inhomogeneity of POS feedstock and magnetic particles during carbonization, there were irregularities in the average pore diameter, specific surface area, and total pore volume changes. However, MPOSB had the highest specific surface area and total pore volume at the carbonization temperature, carbonization retention time, and m(Fe^3+^):m(POS) of 600 °C, 1 h, and 0.1, respectively.

**Figure 4:**
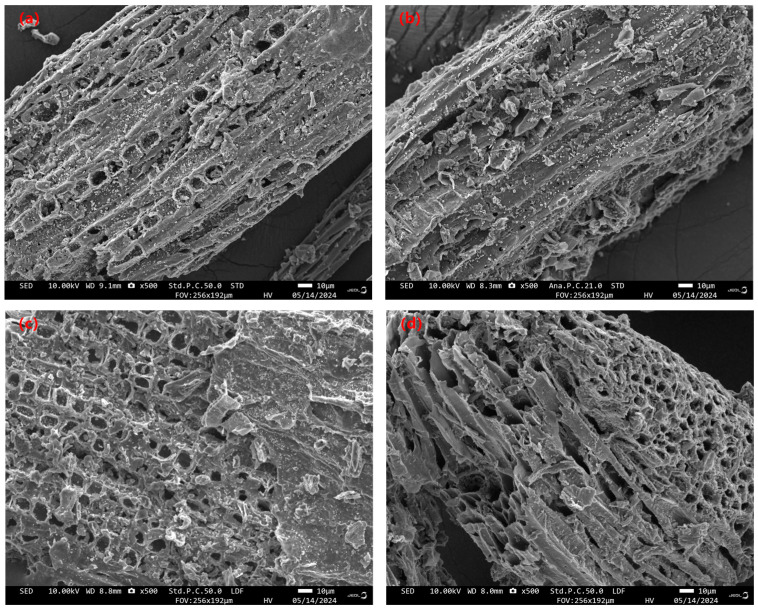
SEM images of MPOSB subjected to different carbonization processes ((**a**–**d**) samples in Table 9).

The SEM images of the MPOSB samples prepared under four different carbonization conditions are shown in Figure 4, where it can be observed that the surface structures of the four samples were irregular, but the distribution of magnetic particles on their surface was relatively uniform. The MPOSB obtained at the m(Fe^3+^):m(POS) ratio of 0.2 had a significantly higher number of magnetic particles distributed on the surface than the sample with the m(Fe^3+^):m(POS) ratio of 0.1. The number of pore spaces in the biochar corresponded to the total pore volume reported in Table 10, and its smaller pore volume was due to the fact that a significant number of magnetic particles occupied part of the pore space. Notably, changes in carbonization temperature did not greatly alter the surface structure of the MPOSB, while the relative content of Fe^3+^ during the impregnation process greatly influenced the aggregation distribution of magnetic particles on the surface of the sample.

**Figure 5:**
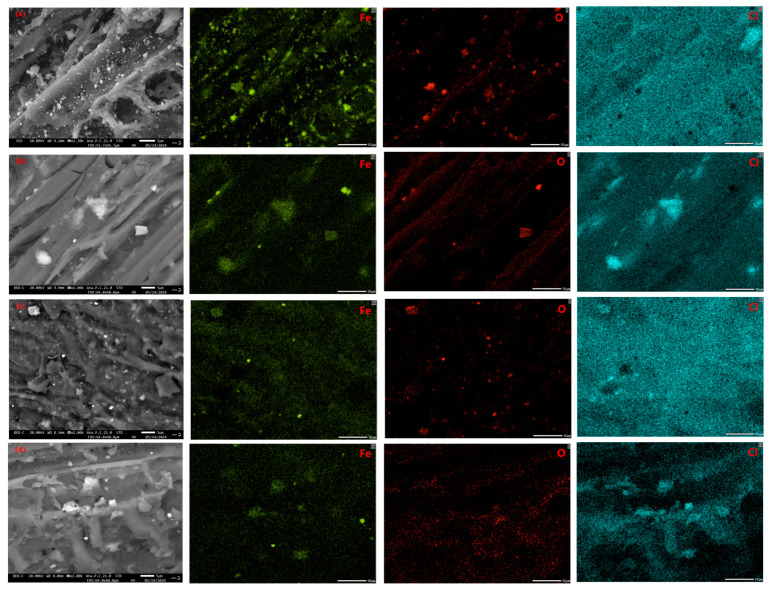
Energy spectra of MPOSB subjected to different carbonization processes ((**a**–**d**) samples in Table 9).

The distribution of iron on the surface of the MPOSB was further determined by using EDS spectroscopy. As shown in Figure 5 for the four carbonization process conditions, the distribution of iron, oxygen, and chlorine on the surface of the MPOSB was found to be consistent with the forms of iron present at different carbonization temperatures, chloritization at 400 °C, and transformation into metal oxides at 600 °C. It was proved that iron content was also one of the reasons for the change in the adsorption properties of the MPOSB.

**Figure 6:**
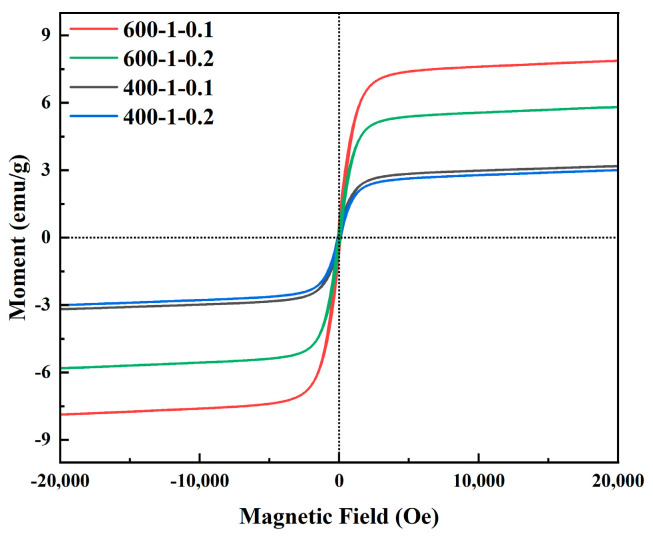
Hysteresis regression lines of MPOSB subjected to different carbonization processes (samples in Table 9).

At room temperature, all four MPOSB types exhibited no hysteresis, and their coercivity and remanent magnetization were 0 (Figure 6). The curves showed saturation magnetization strengths of 7.86, 5.80, 3.18, and 3.00 emu/g, indicating that the prepared magnetic biochar was superparamagnetic and could be recycled with the application of a magnetic field [42]. The curves showed that the MPOSB with m(Fe^3+^):m(POS) = 0.1 had greater magnetization intensity than that of MPOSB with m(Fe^3+^):m(POS) = 0.2. This verified the correct optimization of the m(Fe^3+^):m(POS) process parameter in the model.

### Reusability of the MPOSB

**Figure 7:**
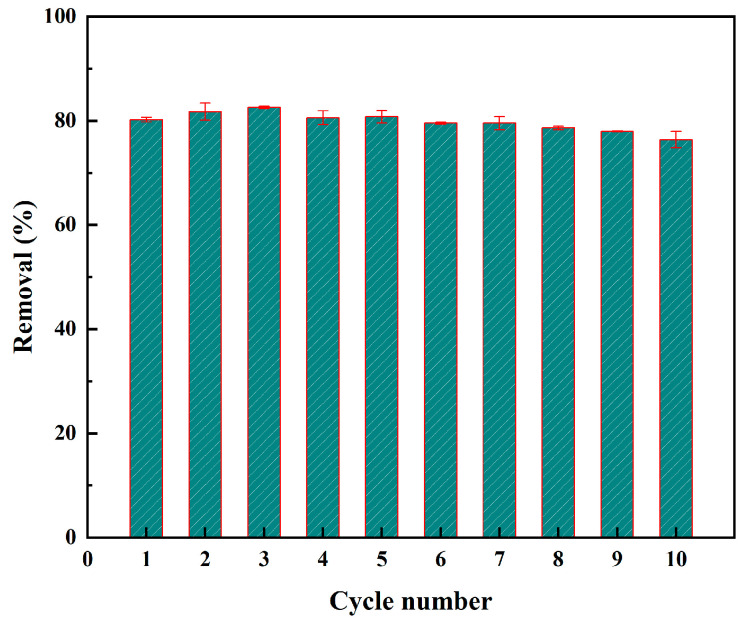
Cycling performance of the MPOSB (under the optimal preparation conditions of carbonization temperature, retention time, impregnation rate, particle size of 600 °C, 1 h, 0.1, and 0.1–0.45 mm, respectively) for MB removal.

The reusability of adsorption materials is crucial in practical applications. Therefore, we evaluated whether the MPOSB retains its high adsorption capacity after undergoing 10 adsorption–desorption cycle experiments. The results showed that after 10 cycles, the adsorption performance of biochar for methylene blue decreased by only 3.84% (Figure 7). This demonstrates that the magnetic biochar prepared by this method exhibits excellent reusability.

## 4. Materials and Methods

### 4.1. Materials

Discarded *Pleurotus ostreatus* substrate (POS) was collected from the edible mushroom cultivation base in Shanxi Agricultural University, Jinzhong, China. Hexahydrate iron (III) chloride (99%), anhydrous ethanol (≥99.7%), and MB dye (1%) were procured from Macklin Co., Ltd. (Shanghai, China). Ultra-pure water was obtained using an ultra-pure water system (Pureforce, Heal Force, Shanghai, China).

### 4.2. Preparation of Magnetic Biochar

*Pleurotus ostreatus* substrate, here used as the raw material, was washed, dried, and ground into powder, which was then sieved to obtain two groups of test materials according to particle size: 0.45–1 mm and 0.1–0.45 mm. Solid powder of FeCl_3_ 6H_2_O (4.821 g) was added to a beaker containing 50 mL of deionized water and continuously stirred until fully dissolved. The sieved POS powder was then added slowly until immersed in the solution. The mass of POS powder added per beaker was 5 g or 10 g, so the ratio of m(Fe^3+^):m(POS) was 1:5 or 1:10. The solution was left to stand at room temperature for 48 h and then filtered, and the resulting materials were vacuum-dried at 105 °C until constant weight.

### 4.3. Characterization of MPOSB

A small amount of dried biochar sample was securely attached to the sample stage using conductive double-sided tape. After approximately 60 s of gold sputtering, the sample’s microstructure and surface elemental composition were observed using a field emission scanning electron microscope (SEM, JSM-IT800, JEOL, Tokyo, Japan). The Brunauer–Emmett–Teller (BET) specific surface area of the magnetic biochar was detected using a specific surface and pore size analysis instrument (3H-2000PS1, BSD, Shenzhen, China). The magnetic hysteresis loops of biochar were recorded on vibrating sample magnetometer (VSM, 8600 Series, Lake Shore Cryotronics, Inc., Woburn, MA, USA) at 25 °C in a magnetic field range from −20,000 to 20,000 Oe. The ultraviolet–visible (UV–vis) absorption spectra were tested with a UV–vis spectrophotometer (UV-2600i, Shimadzu, Beijing, China).

### 4.4. Carbonization Equipment

For the carbonization process, we used a small-scale microwave pyrolysis furnace (CY-OY1100C-S; Hunan Changyi Microwave Technology Co., Ltd., Changsha, China), to which the carrier gas, nitrogen, was supplied from a gas tank.

### 4.5. Adsorption of Methylene Blue

The adsorption of MB was performed in a shaker at 300 rpm and 25 °C for 30 min. A volume of 4 mL of 70 mg/L MB solution was spiked with 0.1 g of biochar. The MB adsorption ability and removal efficiency of biochar was quantified by using Equations (5) and (6), as follows:

(5) $$q_{e}=\frac{\left(C_{0}-C_{eq}\right)V}{m}\times 100,$$

(6) $$R=\frac{{(C}_{0}-C_{eq})V}{m}$$

where *R* (%) and *q_e_* (mg/g) represent the removal percentage and the MB adsorption ability of biochar. *C*_0_ and *C_eq_* are the initial and final concentrations of the MB solution, respectively. *V* (L) is the total volume of MB solution, and *m* (g) is the mass of biochar powder used in the absorption experiment.

### 4.6. Reusability Evaluation

After the adsorption process was complete, the biochar was immobilized on the side of the reaction vessel using a strong magnet. Following magnetic separation, the obtained biochar was washed multiple times with deionized water and anhydrous ethanol. The collected adsorbent was then dried in a vacuum oven at 60 °C for 2 h and used for subsequent adsorption–desorption experiments. To evaluate the stability and reusability of the biochar, 10 regeneration cycles were conducted.

## 5. Conclusions

The P-B design and RSM-CCD method were used to analyze the impact of carbonization temperature, carbonization retention time, and the ratio of m(Fe^3+^) to m(POS) on the adsorption performance of MPOSB during the preparation process. The order of effect significance was as follows: carbonization temperature > m(Fe^3+^) to m(POS) > carbonization retention time. The adsorption of methylene blue under the optimal preparation conditions of carbonization temperature, retention time, impregnation rate, particle size of 600 °C, 1 h, 0.1, and 0.1–0.45 mm, respectively, was 2297.04 μg/g, and the yield was 42.54%. The prepared magnetic biochar was superparamagnetic (Ms: 7.86 emu/g), with a large surface area (37.17 m^2^/g). The RSM was used to establish the fitting equations and 3D surface plots of MPOSB and the three influencing factors, providing a theoretical basis and data support for the preparation of high-quality magnetic biochar from agricultural wastes with carbonization.

## Figures and Tables

**Table 1:** Main parameters of carbonization.

Conditional	Parameter
Carbonization power (w)	700
Carbonization temperature (°C)	400–600
Biomass	*Pleurotus ostreatus* substrate
Material particle size (mm)	0.1–1
Sample weight (g)	5
Carrier gas	N_2_
Carrier gas flow rate (mL/min)	200

**Table 2:** P-B experimental design factors and levels.

Code	Response Variable	Low Level (−1)	High Level (+1)
A	Carbonization temperature (°C)	400	600
B	Carbonization retention time (h)	1	2
C	m(Fe^3+^):m(POS)	0.1	0.2
D	Material particle size (mm)	0.1–0.45	0.45–1

**Table 3:** CCD experimental design factors and levels.

Response Variable	Code	Level		
		−1	0	+1
Carbonization temperature (°C)	A	400	500	600
Carbonization retention time (h)	B	1	1.5	2
M(Fe^3+^):m(POS)	C	0.1	0.15	0.2

**Table 4:** P-B experimental design and response values.

Run	A	B	C	D	E	F	G	H	J	K	L	Y_1_/%	S_1_/μg/g
1	1	−1	1	1	−1	1	1	1	−1	−1	−1	43.1	1108.0
2	1	1	−1	1	1	1	−1	−1	−1	1	−1	40.2	1170.2
3	1	1	1	−1	−1	−1	1	−1	1	1	−1	42.1	1806.5
4	−1	−1	−1	−1	−1	−1	−1	−1	−1	−1	−1	48.2	1833.9
5	1	1	−1	−1	−1	1	−1	1	1	−1	1	40.7	2079.6
6	−1	1	1	1	−1	−1	−1	1	−1	1	1	47.9	577.5
7	−1	−1	1	−1	1	1	−1	1	1	1	−1	50.0	1585.8
8	1	−1	1	1	1	−1	−1	−1	1	−1	1	43.2	1125.9
9	−1	−1	−1	1	−1	1	1	−1	1	1	1	48.0	980.6
10	−1	1	−1	1	1	−1	1	1	1	−1	−1	46.3	822.7
11	−1	1	1	−1	1	1	1	−1	−1	−1	1	48.5	1411.0
12	1	−1	−1	−1	1	−1	1	1	−1	1	1	42.0	2227.1

Note: E, F, G, H, J, K, and L are dummy variables.

**Table 5:** Deviation regression coefficients and significance tests for response values.

Response Value	Response Variable	Bias Regression Coefficient	Standard Error	F-Value	*p*-Value
Yield	A	−3.13	0.0333	8836.00	<0.0001
	B	−0.7333	0.0333	484.00	<0.0001
	C	0.7833	0.0333	525.25	<0.0001
	D	−0.2333	0.0333	49.00	0.0002
Adsorption value	A	192.15	4.77	1623.63	<0.0001
	B	−82.82	4.77	301.61	<0.0001
	C	−124.95	4.77	686.56	<0.0001
	D	−429.92	4.77	8127.81	<0.0001

**Table 6:** CCD experimental design and response values.

Ordinal	Code			Actual Value			Y_2_/%	S_2_/μg/g
	A	B	C	X_1_	X_2_	X_3_		
1	−1	−1	−1	400	1	0.1	48.9	1851.9
2	1	−1	−1	600	1	0.1	42.5	2297.0
3	−1	1	−1	400	2	0.1	49.4	1775.3
4	1	1	−1	600	2	0.1	39.3	2155.0
5	−1	−1	1	400	1	0.2	51.4	1521.4
6	1	−1	1	600	1	0.2	42.6	2246.0
7	−1	1	1	400	2	0.2	50.1	1281.4
8	1	1	1	600	2	0.2	41.2	1826.5
9	−1.68179	0	0	331.82	1.5	0.15	55.1	2292.4
10	1.68179	0	0	668.18	1.5	0.15	40.9	2799.7
11	0	−1.68179	0	500	0.66	0.15	45.3	1273.5
12	0	1.68179	0	500	2.34	0.15	45.0	1613.1
13	0	0	−1.68179	500	1.5	0.066	39.4	1665.7
14	0	0	1.68179	500	1.5	0.234	47.9	1583.2
15	0	0	0	500	1.5	0.15	45.4	1031.5
16	0	0	0	500	1.5	0.15	45.3	1042.7
17	0	0	0	500	1.5	0.15	45.1	1051.3
18	0	0	0	500	1.5	0.15	45.8	1024.5
19	0	0	0	500	1.5	0.15	45.6	1019.7
20	0	0	0	500	1.5	0.15	45.1	1033.6

**Table 7:** ANOVA of MPOSB methylene blue adsorption models.

Source	Sum of Squares	Degree of Freedom	Mean Square	F-Value	*p*-Value
Model	5.159 × 10^6^	9	5.732 × 10^6^	22.77	<0.0001
A	6.362 × 10^5^	1	6.362 × 10^5^	25.27	0.0005
B	6899.57	1	6899.57	0.2741	0.6120
C	1.32 × 10^5^	1	1.32 × 10^5^	5.24	0.0450
AB	7497.0	1	7497.0	0.2978	0.5972
AC	24,742.0	1	24,742.0	0.9829	0.3449
BC	24,299.1	1	24,299.1	0.9635	0.3490
A2	3.949 × 10^6^	1	3.949 × 10^6^	156.89	<0.0001
B2	2.573 × 10^5^	1	2.573 × 10^5^	10.22	0.0095
C2	5.631 × 10^5^	1	5.631 × 10^5^	22.37	0.0008
Residual	2.517 × 10^5^	9	25,110.68		

**Table 8:** ANOVA of MPOSB yield model.

Source	Sum of Squares	Degree of Freedom	Mean Square	F-Value	*p*-Value
Model	292.2	9	33.24	22.4	<0.0001
A	247.02	1	247.02	166.42	<0.0001
B	2.55	1	2.55	1.72	0.219
C	27.83	1	27.83	18.75	0.0015
AB	1.81	1	1.81	1.22	0.296
AC	0.18	1	0.18	0.1213	0.7349
BC	5.684 × 10^−14^	1	5.684 × 10^−14^	3.83 × 10^−14^	1
A2	12.68	1	12.68	8.55	0.0152
B2	0.0695	1	0.0695	0.0468	0.833
C2	5.18	1	5.18	3.49	0.0912
Residual	14084	1.0			

**Table 9:** Optimization and validation of test conditions for MPOSB preparation.

Code	Target	Validation	Experimental Conditions			Y/%	S/μg/g	Desirability
			Carbonization Temperature (°C)	Retention Time (h)	m(Fe^3+^):m(POS)			
a	Adsorption	Model	600	1	0.2	43.582	2171.711	1
		Experiment	600	1	0.2	42.6	2246.0	
b	Yield	Model	400	1	0.2	51.438	1567.584	1
		Experiment	400	1	0.2	51.42	1521.4	
c	X_1_	Model	600	1	0.1	41.027	2146.887	1
		Experiment	600	1	0.1	42.54	2297.04	
d	X_2_	Model	400	1	0.1	48.283	1765.210	1
		Experiment	400	1	0.1	48.86	1851.96	

**Table 10:** Specific surface area values of MPOSB under different carbonization processes.

Samples in Table 9	a	b	c	d
Specific surface area (m^2^/g)	7.073	8.5819	37.170	4.5655
Total pore volume (cm^3^/g)	0.0147	0.0097	0.0304	0.0027
Average pore size (nm)	8.313	4.521	3.2715	2.3656

## Data Availability

The datasets generated during and/or analyzed during the current study are available from the corresponding author upon reasonable request.
